# Adherence to in-person and app-based exercise for patients with hip or knee osteoarthritis; secondary analyses from a randomized controlled trial

**DOI:** 10.1007/s00296-025-05967-4

**Published:** 2025-08-26

**Authors:** Lars Martinsen, Nina Østerås, Tuva Moseng, Anne Therese Tveter

**Affiliations:** 1 Division of Rheumatology and Research, Center for Treatment of Rheumatic and Musculoskeletal Diseases (REMEDY), Diakonhjemmet Hospital, Postboks 23 Vinderen, 0319 Oslo, Norway; 2 Department for Interdisciplinary Health Sciences, Faculty of Medicine, University of Oslo, Oslo, Norway; 3 Department of Rehabilitation Science and Health Technology, Faculty of Health Sciences, Oslo Metropolitan University, Oslo, Norway

**Keywords:** Digital health, Telemedicine, Osteoarthritis, Treatment adherence and compliance, Exercise therapy

## Abstract

**Background:**

Exercise is recommended for patients with hip/knee osteoarthritis (OA), however, adherence to exercise tends to be poor. This study aimed to (1) compare in-person versus app-based adherence to exercise therapy, (2) identify patient characteristics associated with adherence, and (3) examine associations between adherence and change in disease-specific outcomes in hip/knee OA patients.

**Method:**

Data were collected as part of a randomized controlled trial. In-person physiotherapy treatment involved supervised exercise therapy twice weekly for 6 weeks, complemented by an additional weekly home-exercise session. The app group received an individually tailored exercise program in the Virtual Training-app for 6 weeks and were advised to exercise three times weekly. Adherence was recorded as number of exercise sessions attended. Patient characteristics were reported at baseline. Changes in disease-specific outcomes were evaluated at 6-weeks follow-up using Hip/Knee Injury and Osteoarthritis Outcome Score. Associations were assessed using multiple logistic and linear regression analyses.

**Results:**

In total 68 patients, 34 in each group were included (63 years, 69% female). The odds of adherence were 4.2 times higher when exercise was supervised by physiotherapists (95% CI 1.5, 12.4; p = 0.008). High fatigue was associated with lower adherence (OR 0.8, p = 0.049), whereas higher education (OR 5.2, p = 0.02) and self-efficacy (OR 3.2, p = 0.008) were associated with higher adherence. No significant associations were found between disease-specific outcomes and adherence.

**Conclusion:**

App-based exercise did not improve adherence compared to supervised exercise. Apart from fatigue, education, and self-efficacy, no factors were found to influence adherence to exercise in hip/knee OA patients.

**Trial registration:**

ClinicalTrials.gov (NCT04767854).

## Introduction

Exercise therapy is essential in treatment of hip and knee osteoarthritis (OA) [1]. To achieve the desired physiological effect, exercise programs should follow the principles of progressive overload according to FITT (frequency, intensity, time, type) [2]. The American College of Sports Medicine’s recommendation of 2–3 exercise sessions per week as exercise frequency [3] are often used in osteoarthritis management programs (OAMPs), typically employing exercise for at least 6 weeks [4, 5]. Although previous research has emphasized the importance of adhering to exercise to sustain the benefits [6], adherence is often shown to be poor [7, 8] and with unclear relation to clinical outcomes [9].

Digital health technologies, like apps, are increasingly used as a cost-efficient alternative to in-person delivery of OAMPs to provide access to recommended treatment. We have previously conducted a non-inferiority RCT evaluating the effectiveness of a generic exercise app following the recommended exercise frequency in the management of patients with hip or knee OA, which are submitted elsewhere. The results were inconclusive regarding non-inferiority of the exercise app, however with a tendency towards the app being as effective as standard physiotherapy care. A factor that may influence the result of the RCT is exercise adherence. In addition to be effective tools in providing exercise therapy to patients, apps have been hypothesized to enhance adherence to exercise [10]. Apps may offer features that potentially can improve adherence to exercise in OAMPs, such as audio and video instructions, automated feedback for patients and physiotherapists, motivational messages, and opportunities for digital booster sessions [11]. However, previous research has found digital interventions to have little effect on adherence to exercise therapy in musculoskeletal disorders, suggesting the need for further research [12, 13]. Furthermore, previous research on factors associated with adherence has found variability in adherence to be poorly explainable [14]. Thus, it is unclear which demographic and clinical characteristics may influence adherence. To address the need for more knowledge on digital interventions' impact on adherence, and factors associated with being adherent, we conducted exploratory secondary analyses of a randomized controlled trial (RCT) to: 1) compare the adherence to exercise therapy delivered in-person or through an exercise app, 2) identify patient demographics and clinical characteristics associated with exercise adherence, and 3) examine whether changes in disease-specific outcomes were associated with adherence to exercise in patients with hip or knee OA.

## Methods

### Design and setting

This study included secondary analyses of data from a two-armed, parallel, non-inferiority RCT, pre-registered at ClinicalTrials.gov (NCT04767854). The study protocol is published and results on the primary outcome have been submitted elsewhere [15]. The study was conducted in outpatient physiotherapy clinics in primary care in Norway. All patients provided written informed consent before participating in the study.

### Patients, recruitment and randomization

The patients were recruited by physiotherapists at 8 clinics between June 2021 and December 2023. Eligibility criteria included people aged 18 years or older with activity-related hip or knee joint complaints or symptoms corresponding to hip or knee OA, and access to a smartphone/tablet. Patients were excluded if they had a neurological disorder, contraindication to physical activity, total hip or knee replacement in the index joint, inflammatory rheumatic diseases, malignant illness or other major conditions that restricted the ability to adhere to the recommended treatment, or did not understand the Norwegian language. Stratified by clinic and index joint, patients were randomly allocated in a 1:1 ratio with a block size of 10 to either in-person or app-based treatment. Concealed, opaque envelopes with group allocation were prepared in advance based on a digital randomization list [16]. Blinding of physiotherapists and patients was not possible due to the nature of the intervention.

### Intervention

The intervention was based on principles outlined in AktivA, a Norwegian OAMP [17]. The program includes patient education and exercise therapy in line with international recommendations [1]. The patient education included information on signs and symptoms, risk factors, weight control, etc. The exercise therapy included exercises to improve strength, balance, and functional stability. No specific distinction between hip and knee OA was included in the patient education. Distinction between the patient groups regarding exercises were made on the discretion of the physiotherapist. Prior to randomization, baseline data were collected. Patients were then randomized to 6 weeks of individually tailored exercise therapy, either in-person at the physiotherapy clinic (standard treatment group) or through the Virtual Training (VT) app (experimental treatment group). The VT solution is a generic exercise app with a web interface for the physiotherapist and an app for the patient.

In the standard treatment group, patients consulted their physiotherapist twice weekly for exercise therapy and were encouraged to undertake an additional weekly home exercise session. Program adjustments were made during supervised sessions. The experimental treatment group was advised to conduct their individualized digitally delivered program three times per week. The physiotherapists monitored the progression in the web interface once weekly, based on self-report from patients after each session. If the self-reported effort fell below 60% or exceeded 85%, or if self-reported pain scores were ≥ 5, the physiotherapists were instructed to reach out to modify the program.

Both groups were instructed to complete a total of 18 sessions throughout the intervention period. No restrictions were set regarding additional exercise sessions. The physiotherapists did not contact patients who failed to adhere to the advised number of exercise sessions.

### Outcome measures

Patient-reported outcomes were collected at baseline and 6-week follow-up using an encrypted web-based questionnaire solution (Nettskjema) delivered by the University of Oslo.

Session attendance was recorded by the physiotherapists in the standard treatment group and from the VT app in the experimental treatment group. Home-based sessions in the standard treatment group were self-reported in the 6-week follow-up questionnaire. Adherence was defined as performing exercise therapy sessions and/or home exercises ≥ 2 times per week for 6 weeks.

Patient demographics and clinical characteristics are presented in Table 1 and are described in detail in the protocol article [12]. In short, the characteristics include patient-specific characteristics as age, sex, BMI, living status, educational level, work level, and disease history. Clinical characteristics include pain, fatigue, disease activity, comorbidities, and pain medication use. Patient-reported outcome measures include Hip/Knee Injury and Osteoarthritis Outcome Score (H/KOOS) for measures of pain, function, and health related quality of life, EQ-5D for measures of health-related quality of life, Exercise Health Belief Questionnaire for measures of exercise self-efficacy, Arthritis self-efficacy scale (ASES) for measures of disease-specific self-efficacy, and International Physical Activity Questionnaire (IPAQ) for measures of weekly physical activity level. Disease-specific outcomes were derived from the HOOS and KOOS [18, 19].

**Table 1:** Baseline characteristics of total sample

	Total sample (*n* = 68)
Age, years, mean (SD)	63 (11)
Females, n (%)	44 (69)
BMI, kg/m^2^, mean (SD)	28 (6)
Living alone, n (%)	16 (24)
Education at college/university level, n (%)	43 (64)
Smoking (yes), n (%)	3 (4)
*Work status*, n (%)	
In paid work, fully/partly	29 (43)
Sick leave, fully/partly	4 (6)
Age retirement	30 (45)
Receiving disability pension	4 (6)
*Most painful joint*, n (%)	
Hip (left or right)	21 (31)
Knee (left or right)	46 (69)
*Number of other painful joints* (range 0–9)^a^, n (%)	
0	10 (15)
1–4	52 (78)
5–9	5 (7)
*Symptom duration*, n (%)	
Less than a year	7 (11)
1–5 years	34 (52)
6–10 years	11 (17)
More than 10 years	14 (21)
Disease activity last week, mean (SD) (NRS 0–10, 0 = no disease activity)	5 (2)
Pain last week, mean (SD) (NRS 0–10, 0 = no pain)	5 (2)
Fatigue last week, mean (SD) (NRS 0–10, 0 = no fatigue)	4 (2)
Comorbidities (yes)^b^, n (%)	40 (62)
Current pain medication use (yes), n (%)	52 (78)
30-s chair-stand test (30 CST) (number of repetitions, higher number = better performance)	14 (4)
Patient-Specific Function Scale (PSFS) (NRS 0–10, 10 = no problem)	4 (3)
Health-related quality of life (EQ-5D-5L VAS) (0–100, 100 = best health)	65 (18)
Mental health – Hopkins Symptom Checklist 5 (HSCL-5) (1–4, 1 = best mental health)	1.5 (0.5)
*Hip/knee injury and osteoarthritis outcome score* (H/KOOS) (0–100, 100 = no problems)	
Pain	53.8 (17.4)
Symptoms	47.7 (14.5)
Activities of Daily Living	64.1 (20.1)
Sports and recreational function	36.3 (20.0)
Quality of life	40.4 (18.0)
Self-efficacy – Exercise Health Belief Questionnaire (0–100, 100 = strongest belief)	78.5 (12.7)
Self-efficacy, sum score (4–20)	14.6 (3.5)
Barriers, sum score (3–15)	12.1 (2.1)
Benefits, sum score (5–25)	20.5 (2.8)
Impact on arthritis, sum score (8–40)	32.2 (4.8)
*Self-efficacy – Arthritis self-efficacy scale* (0–4, 4 = best self-efficacy)	
Pain	2.3 (0.8)
Symptoms	2.4 (0.8)
International Physical Activity Questionnaire-Short Form (IPAQ-SF) (MET-minutes/week) (median (IQR))	2186 (899–3600)

*SD*, standard deviation; *IQR*, interquartile range; *NRS*, numeric rating scale; *VAS*, visual analog scale ^a^Hip, knee, ankle, hand/fingers, others; ^b^hypertension, coronary disease, allergy, sciatica, cerebrovascular accident, cancer, neurological disorder, diabetes, metabolic disorder, psychiatric disorder, renal disease, hepatic disease, gastrointestinal disease, hematologic disorder

### Statistical analyses

STATA version 17 was used for statistical analysis, and the level of significance was set to *p* < 0.05. The estimated sample size calculation for the primary analysis in the main study was 156 patients, 78 in each arm [15]. However, only 68 patients were recruited, 34 in each arm, due to slow recruitment and time constraints. The difference in proportion of adherent patients between the experimental and standard treatment groups is presented descriptively and analysed with logistic regression adjusted for age and sex.

For the second and third objectives of the current study, analyses were conducted with data from the two groups combined, irrespective of group allocation.

Associations between adherence and patient demographic and clinical characteristics were analysed using multiple logistic regression. Separate analyses of each characteristic were chosen due to the limited sample size. Analyses were adjusted for age, sex, and group allocation. The difference between adherent and non-adherent patients on disease-specific outcomes was analysed using multiple linear regression, adjusted for age, sex, and group allocation.

### Patient and public involvement

Two patient research partners were involved in the planning of the study, including writing the grant application and designing the intervention.

## Results

Baseline characteristics of the 68 included patients are presented in Table 1.

The median number of exercise sessions attended for the total sample was 14 (IQR 10–18), ranging from 0 to 55 sessions. In the standard treatment group, the median number of supervised exercise sessions was 10 (IQR 7–12), with a range from 0 to 17 sessions, while the median number of home exercise sessions was 3 (IQR 0–12), ranging from 0 to 42 sessions. Combining in-person and home-based sessions, the standard treatment group performed a total of 13 (IQR 11–21) sessions over 6 weeks. In the experimental treatment group, the median number of app-registered exercise sessions was 14 (IQR 9–17), ranging from 0 to 21.

**Fig. 1:**
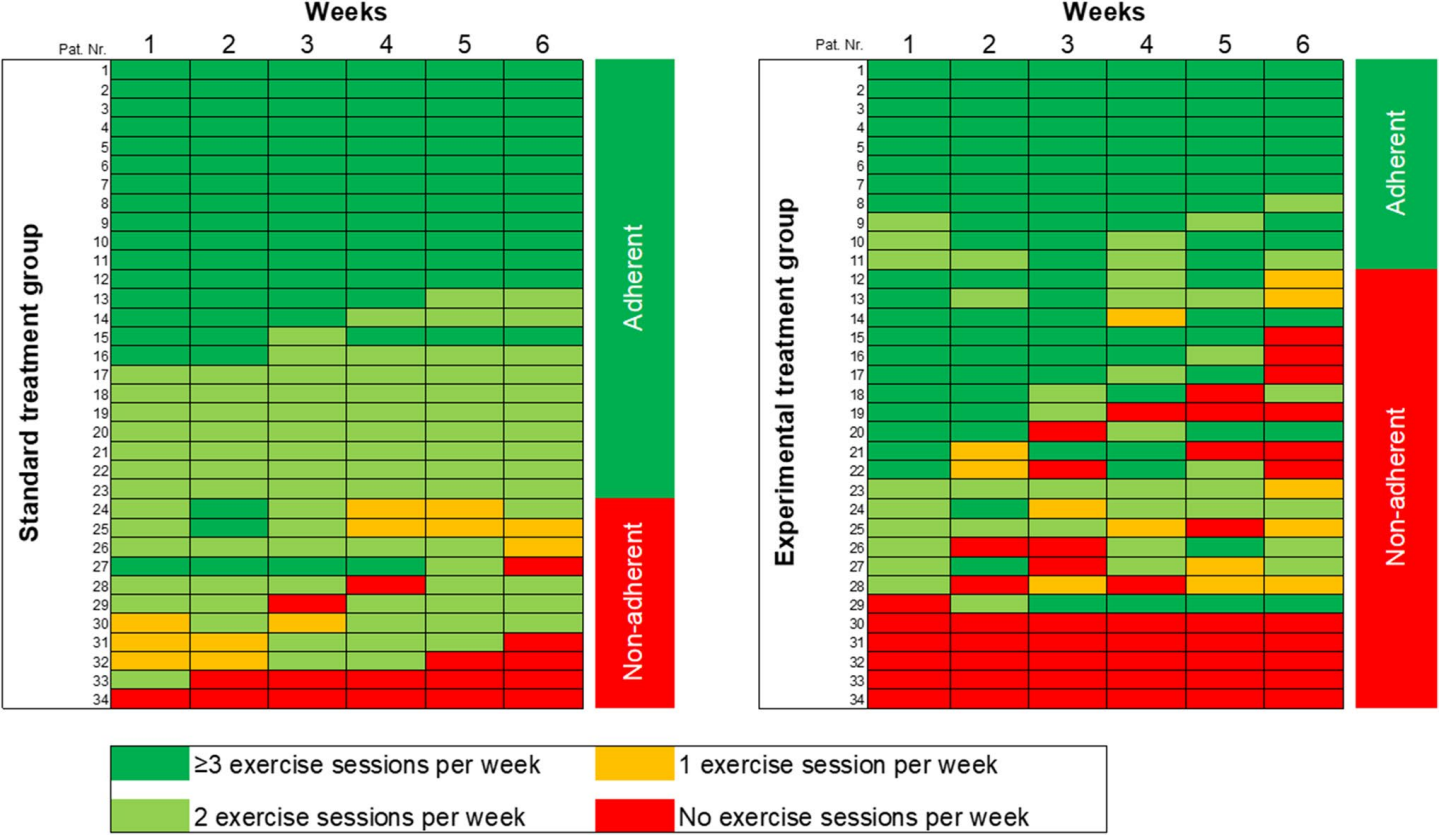
Adherence to exercise per week

Overall, 34 (50%) patients were categorized as adherent, with 23 (68%) in the standard treatment group and 11 (32%) in the experimental treatment group (Fig. 1). The odds ratio (OR) for adherence was 4.2 times higher (95% CI 1.5, 12.4; *p* = 0.008) in the standard treatment group compared to the experimental treatment group. 

Baseline characteristics and odds ratio of adherent versus non-adherent patients are presented in Table 2. Patients with high levels of fatigue at baseline had lower odds of being categorized as adherent [OR 0.8 (95% CI 0.6, 0.99, p = 0.049)]. Opposite, patients with college or university education [OR 5.2 (95% CI 1.3, 20.7 p = 0.02)] and higher levels of self-efficacy for symptom management had higher odds [OR 3.2 (95% CI 1.4, 7.7, p = 0.008)] of being adherent..

**Table 2:** Baseline characteristics of adherent versus non-adherent patients and odds ratio (OR)of being adherent to an exercise program, adjusted for age, gender, and intervention group*

	Non-adherent (*n* = 34)	Adherent (*n* = 34)	OR (95% CI)	p
Age, years, mean (SD)	60 (11)	64 (9)	1.04 (0.99, 1.10)	0.10
Females, n (%)	23 (70)	21 (68)	0.82 (0.25, 2.74)	0.75
BMI, kg/m^2^, mean (SD)	28 (6)	28 (7)	0.97 (0.86, 1.09)	0.57
Living alone, n (%)	7 (21)	9 (27)	1.20 (0.30, 4.78)	0.80
Education at college/university level, n (%)	19 (56)	24 (73)	5.19 (1.30, 20.72)	0.02
Smoking (yes), n (%)	1 (3)	2 (6)	2.01 (0.15, 27.15)	0.60
*Work status*, n (%)				
In paid work, fully/partly	15 (44)	14 (42)	2.63 (0.61, 11.44)	0.20
Most painful joint, n (%)				
Hip (left or right),_ref_	14 (41)	7 (21)		
Knee (left or right)	20 (59)	26 (79)	2.01 (0.63, 6.49)	0.24
*Number of other painful joints* (range 0–9)^a^, n (%)				
0,_ref_	5 (15)	5 (15)		
1–4	26 (76)	26 (79)	1.41 (0.31, 6.39)	0.65
5–9	3 (9)	2 (6)	0.92 (0.09, 9.44)	0.95
Symptom duration, n (%)				
Less than a year,_ref_	4 (12)	3 (9)		
1–5 years	18 (55)	16 (49)	0.84 (0.14, 4.98)	0.84
6–10 years	4 (12)	7 (21)	1.41 (0.17, 11.75)	0.75
More than 10 years	7 (21)	7 (21)	0.79 (0.10, 6.01)	0.82
Disease activity last week, mean (SD) (NRS 0–10, 0 = no disease activity)	5 (2)	5 (2)	0.90 (0.67, 1.20)	0.48
Pain last week, mean (SD) (NRS 0–10, 0 = no pain)	5 (2)	5 (2)	0.81 (0.60, 1.09)	0.16
Fatigue last week, mean (SD) (NRS 0–10, 0 = no fatigue)	5 (2)	3 (2)	0.76 (0.58, 0.99)	0.049
Comorbidities (yes)^b^, n (%)	21 (62)	19 (61)	0.96 (0.31, 2.97)	0.95
Current pain medication use (yes), n (%)	28 (82)	24 (73)	0.64 (0.17, 2.45)	0.51
30-s chair-stand test (30 CST)	14 (3)	15 (4)	1.17 (0.99, 1.40)	0.07
Patient-Specific Function Scale (PSFS) (NRS 0–10, 10 = no problem)	4 (3)	4 (3)	1.02 (0.83, 1.26)	0.82
Health-related quality of life (EQ-5D-5L VAS) (0–100, 100 = best health)	62 (20)	68 (14)	1.02 (0.99, 1.06)	0.20
Mental health – Hopkins Symptom Checklist 5 (HSCL-5) (1–4, 1 = best mental health)	1.6 (0.6)	1.5 (0.4)	0.93 (0.30, 2.88)	0.90
*Hip/knee injury and osteoarthritis outcome score* (H/KOOS) (0–100, 100 = no problems)				
Pain	52.6 (17.9)	55.0 (17.1)	1.00 (0.97, 1.03)	0.88
Symptoms	47.2 (17.5)	48.2 (11.1)	0.99 (0.95, 1.03)	0.57
Activities of Daily Living	62.5 (21.0)	65.7 (19.4)	1.01 (0.98, 1.03)	0.67
Sports and recreational function	35.1 (19.2)	37.5 (21.0)	1.01 (0.98, 1.04)	0.53
Quality of life	38.6 (18.3)	42.2 (17.8)	1.01 (0.98, 1.04)	0.54
Self-efficacy – Exercise Health Belief Questionnaire (0–100, 100 = strongest belief)	78.5 (10.1)	78.5 (15.1)	1.11 (0.94, 1.30)	0.21
Self-efficacy, sum score (4–20)	14.2 (3.2)	15.0 (3.7)	1.01 (0.96, 1.05)	0.70
Barriers, sum score (3–15)	12.0 (2.0)	12.1 (2.3)	0.93 (0.70, 1.23)	0.61
Benefits, sum score (5–25)	20.6 (3.1)	20.3 (2.5)	1.03 (0.84, 1.28)	0.74
Impact on arthritis, sum score (8–40)	31.7 (5.2)	32.8 (4.3)	1.04 (0.02, 1.17)	0.55
*Self-efficacy – Arthritis self-efficacy scale* (0–4, 4 = best self-efficacy)				
Pain	2.3 (0.8)	2.2 (0.9)	0.79 (0.39, 1.57)	0.49
Symptoms	2.2 (0.7)	2.7 (0.8)	3.22 (1.36, 7.65)	0.008
International Physical Activity Questionnaire-Short Form (IPAQ-SF) (MET-minutes/week) (median (IQR))	2182 (779–3732)	2236 (1095–3521)	0.99 (0.99, 1.00)	0.17

*SD*, standard deviation; *IQR*, interquartile range; *NRS*, numeric rating scale; *VAS*, visual analog scale; *ref*, reference value *Odds of being adherent evaluated with multiple logistic regression adjusted for age, sex and primary group allocation (experimental treatment group or standard treatment group) for each variable separately; ^a^hip, knee, ankle, hand/fingers, others; ^b^hypertension, coronary disease, allergy, sciatica, cerebrovascular accident, cancer, neurological disorder, diabetes, metabolic disorder, psychiatric disorder, renal disease, hepatic disease, gastrointestinal disease, hematologic disorder

No significant differences in disease-specific outcomes were found between adherent versus non-adherent patients (Table 3).

**Table 3:** Between-group differences in mean change scores for HOOS/KOOS combined for non-adherent vs. adherent patients (n = 53)*

	Non-adherent patients (n = 23)				Adherent patients (n = 30)				Between-group difference, baseline to 6-week follow-up		p
	Baseline		6-week follow-up		Baseline mean (SD)		6-week follow-up mean (SD)		Mean	(95% CI)	
	Mean	(SD)	Mean	(SD)	Mean	(SD)	Mean	(SD)			
Pain	51.8	17.5	59.6	21.2	57.0	16.3	61.3	17.1	− 2.3	(− 9.4, 4.8)	0.52
Symptoms	44.8	20.2	47.8	20.9	48.3	10.9	54.4	14.0	6.3	(− 1.1, 13.7)	0.09
ADL	60.6	21.3	67.1	21.4	67.9	17.5	69.9	17.7	− 4.1	(− 11.1, 2.9)	0.25
Sports/rec^b^	34.1	17.9	40.2	24.0	38.6	20.8	38.2	21.0	− 7.0	(− 16.4, 2.4)	0.14
QoL	37.0	18.8	42.4	20.2	44.3	16.7	46.6	16.5	− 3.3	(− 3.4, 30.0)	0.12

*ADL*, activities of daily living; *Sports/rec*, sport and recreational function; *QoL*, quality of life ^a^Adjusted for age, sex and primary group allocation (experimental treatment group or standard treatment group); ^b^1 missing response among the adherent patients (n = 29); *results only for patients reporting HOOS/KOOS at 6-week follow-up

## Discussion

This study aimed to compare adherence to exercise therapy delivered in-person and through an exercise app in patients with hip or knee OA. Furthermore, the study aimed to identify characteristics of patients categorized as adherent and evaluate the impact of adherence on disease-specific outcomes. The results showed that patients receiving in-person supervised physiotherapy treatment had 4.2 higher odds of adherence compared to patients utilizing the exercise app. Higher baseline fatigue negatively impacted adherence, while higher levels of education and self-efficacy positively influenced adherence, regardless of group allocation. Being adherent appeared not to influence disease-specific outcomes.

Our results did not show higher adherence when using an exercise app compared to in-person treatment, contrasting previous studies reporting up to 68% of patients maintaining high adherence in a digital OAMP [20]. They also contrast the results of a systematic review of 10 RCTs in various conditions, finding that the addition of digital interventions increased exercise adherence in 70% of the studies [21]. However, the latter review by Lang et al. reviewed digital interventions as an adjunct to traditional therapy, not as a stand-alone intervention as in our study. Although there was an option for support from the physiotherapist in the present study, this was not intended as a blended intervention. Thus, it may appear as there could be a difference between apps aimed for self-support in contrast to apps intended for blended use. Further, the higher odds of being adherent when receiving in-person treatment, as observed in our study, might be explained by a commitment to attend sessions when a physiotherapist was present. Creating a similar perception of commitment to undertake app-based exercises may increase adherence in digitally delivered OAMPs. Utilization of text messaging has previously been evaluated, with beneficial effect on adherence [22], and further assessments of how technological features can enhance commitment, and potentially adherence, should be pursued.

A common issue regarding digital interventions, and apps specifically, is high dropout rates [23]. It has in previous research been called for a need for “a science of attrition” [24], evaluating why patients becomes non-adherent to the new technology. Evaluation of the usability of the app conducted in the primary non-inferiority RCT indicated good usability, similar to observations in other exercise apps [25]. This implies that the perceived ease of use of the app is high, making it is less likely that there are issues with the app itself that causes non-usage. When adherence is measured by app engagement, as in our study, the low adherence may reflect a lack of engagement with the app rather than unwillingness to perform exercises. Lack of engagement could be related to an experience of lack of perceived usefulness. Our results showed that the majority of the non-adherent patients stopped using the app after 2–3 weeks, possibly implying that they had learned the exercises and no longer needed to engage with the app. Similar results have been seen in research evaluating long-term adherence to digitally provided exercises in other patient populations. In a study by Paul et al. highest adherence was observed the first four weeks, with a steady decline throughout the observation period [26]. If the patients do not experience that the app provides any additional benefit, it is likely that they will discontinue its use. This obstacle may be surpassed by providing elements in the app that could motivate for further use, such as elements of gamification [27]. However, in the present app, few gamification elements were included, potentially contributing to non-adherence. Further, the results also illustrate how different measures and definitions of adherence could influence the result [28]. In the present study, utilizing another measure than frequency, a different definition, or an objective measure of exercise adherence, like an accelerometer, could have provided different results. Thus, there is a need for standardization of how to measure adherence.

The app used in this study allowed for personalization and individual tailoring of the exercise. These features are previously found to positively influence adherence to apps, together with individual factors such as age, healthy BMI, and a positive attitude towards technology [29]. Except for fatigue, education, and self-efficacy, no other baseline factors were significantly associated with adherence in our study, hampering the ability to identify patients who may benefit from a digital intervention. However, as these results are only correlational, and do not include causality, they should be interpreted with caution in relation to their ability to explain individual factors that may influence adherence to an app. To effectively stratify the use of digital health technology guided by individual factors, further research is needed.

No significant difference was observed regarding disease-specific outcomes between adherent and non-adherent patients. Despite this, it appears as there was a tendency towards a higher treatment effect in the non-adherent group. This is a somewhat surprising result, given the assumed influence of adherence on outcomes, although the influence of complying with ACSM recommendations recently has been questioned [30]. However, it should be noted that the results may be influenced by the initial baseline difference between the groups. As the non-adherent group initially started at a lower level, the result may be viewed as they are catching up the difference against the adherent group. Although our result on disease-specific outcomes should be interpreted with caution, it could be viewed as being in line with a recent published systematic review finding lack of a clear association between adherence and the effect size for improvement in pain and physical function [9]. Similar results are found in the study by Kiadaliri et al. [20], which irrespective of level of adherence find uncertain clinically relevant improvements in clinical outcomes. As is discussed in the review, exercise dose is a combination of several factors, not only adherence. Thus, it may be other factors, such as the level of intensity, that may have a greater influence on our result. However, as it in our study has not been evaluated to what degree other elements of the ACSM recommendations has been met, we cannot conclude on what causes the improvement in the two groups.

The pragmatic design and primary care setting represent strengths of our study. Limitations include the small sample size, which raises the risk of chance findings and limits generalizability, thus the results should be interpreted with caution. The small sample size also affects the statistical power of our analysis. As well, the lack of blinding of both patients and therapists poses a risk of bias. However, this is a difficult issue to overcome due to the nature of the intervention, and is an issue recognized in RCTs assessing nonpharmacological treatments [31]. Furthermore, the short intervention period restricts our ability to assess long-term implications.

## Conclusion

In conclusion, the introduction of an exercise app did not improve adherence compared with in-person exercise therapy. Except for fatigue, education and self-efficacy, no factors seemed to influence adherence to exercise in patients with hip/knee OA. Clear knowledge of benefits of implementing digital interventions into clinical practice remains challenging, with a lack of clear guidance on which patients might benefit the most.

## Data Availability

The datasets are available from the corresponding author upon reasonable request.

## Declarations

### Congress abstracts

No part of this manuscript has previously been published in congress abstracts.
